# On the Phenomena, Causes, and Treatment of Sea-Sickness

**Published:** 1808-04

**Authors:** Edward Miller

**Affiliations:** New York


### *On the Phenomena, Causes, and Treatment of Sea-Sickness.* By Edward Miller, M. D. *of New York.*

THIS disease affects most persons on their first going to sea. It is of various degree and duration in different instances; frequently slight and transient; sometimes severe, protracted, and excruciating. In general, it continues only for the first day or two of a voyage, produces little trouble or confinement, and is attended with no danger. In a few cases it begins at the first moment of embarkation, harrasses the patient with incessant tortures for weeks and months, or at least, recurs with violence at every return of bad weather, and only releases him from his sufferings at the end of the voyage. It has likewise happened, on some occasions, that the symptoms of sea-sickness have not disappeared even on the arrival of the vessel in port, and the patient's going ashore. And examples have not been wanting of such derangement of the the system, by the violence and obstinacy of this disease as gradually to induce fever of the worst kind, attended with loss of all retentive power of the stomach, and terminating in death.

Sea-sickness is more apt to occur in the open sea, where the waves have an extensive and uninterrupted course of motion, than in gulphs, bays, channels and rivers. It is chiefly troublesome when the sea is much agitated by wind. The vibrating motion of a vessel, from stem to stern, and from stern to stem, which is called *pitching*, or that from one side to another, called *rolling*, produces the severest degrees of giddiness and sickness. These motions are observed when the vessel is going directly before the wind, or when a calm suddenly succeeds a storm, and not when the wind blows obliquely, or on the *quarter*, for then the succussion which the ship undergoes is much diminished.

In small vessels, on which the slightest movement of the waves makes an impression, this disorder is more likely to take place than in very large ones, such as ships of war, or merchantmen of great burden, deeply laden, which, comparatively, undergo little disturbance. It has been also observed, that where habit produces accommodation to the motions of one vessel, removal to another, whether from a larger to a smaller, or from a smaller to a larger, will sometimes again awaken the disease.

Aged persons are seldom affected with the disease, in comparison of those at the youger and middle periods of life. Those of a dark complexion, in general, suffer less than such as are fair; and infants are commonly altogether exempted.

As a description and an example of the sufferings sometimes endured from this disease, the following account is given, by a medical gentleman, of his own case, in the voyage of the embassy from the King of Great-Britain to the Emperor of China. "He felt," he said, " at first, a sickness in his stomach, followed by a retching, when he threw up whatever he had taken into it; then green, and afterwards yellow bile; to which succeeded a thick, mucilaginous, insipid fluid, which he considered to be the gastric juice; and, lastly, grumous blood. Before he vomited the last, he felt a sensation as if his stomach were twisting together, and which motion, he supposed, produced the hæmorrhage. Had the blood proceeded from the lungs, he judged it would have been spumous, or mixed with air-bubbles (and florid). He felt constantly a nausea in his mouth; mouth; his salivary glands swelled, and the saliva became thickened and vitiated. His mind grew indifferent to all things, either past or future, and even to his existence. Regret and hope were equally extinct within his breast. His head felt light and sore, and as if its sutures were separated from each other. It likewise ached; and he had alternate sensations of violent heat and chilling cold. He thought he felt the inversion of the peristaltic motion, and its actual tendency upwards from the intestines to the mouth. Whatever he swallowed he returned, with no alteration of it in the stomasch. The bare mention of food, solid or liquid, was loathsome to him."*

### Causes of Sea-Sickness.

Sea-sickness † begins with giddiness and vertigo, which not only demand attention in describing the order of the symptoms, but likewise afford a clue to trace the nature and causes of the disease. This species of vertigo originates from disordered action of the organ of vision, produced by the instability and unaccustomed movements of all objects upon the water. That such is the cause of it, is proved from its being excited in some people, though in a less degree, by gazing on the fluctuations of a river (provided no fixed objects appear within the sphere of distinct vision,) or by the sight of a large revolving wheel, while the vertiginous persons themselves are perfectly at rest, and, by shutting their eyes, can instantly arrest this troublsome sensation. Another proof that vertigo may arise from the effects of the instability and indistinctness of visible objects on the eyes, is derived from our dependence upon the steadiness of such objects in walking and in balancing the body. We constantly determine the distances of the objects which we approach by our eyes, and, by observing their perpendicularity, regulate our own: hence no one who is hood-winked can walk in a straight line for an hundred steps together. And when children are learning to walk, it is easy to observe the efforts they make to adjust their perpendicularity by surrounding objects, and how instantaneously they fall, when either their attention

* Staunton's Account of Lord Macartney's Embassy to China, vol. i. p. 145 and 146.

† In this inquiry into the causes of sea-sickness, I have adopted the theory of Dr. Darwin. I have also made use of several of his facts and illustrations, as they are the most familiar and apposite of any which are now within my reach. [See *Zoonomia, vol.* i *sect.* 20.]

attention is unexpectedly called off from this adjustment, or when an object which had caught their eyes, and had been hitherto stationary, is made to undulate. This power of balancing the body by the view of surrounding objects is acquired with difficulty, maintained solely by habit, and may be readily impaired or destroyed by disuse; for persons who have been long confined to bed are found to reel and stagger in their first attempts to walk, and only by patient endeavours recover their former steadiness. The principle of our dependence upon vision in balancing the body by external objects, and of the tendency to vertigo whenever that sense is impaired by disturbance or disease, is still further illustrated by the vertiginous sensations which often affect elderly persons when they begin to suffer dimness of sight, and which are frequently relieved by the use of spectacles, or, at length, by acquiring the habit of adjusting perpendicularity by objects less distinctly seen.

That distinctness of visible objects which is requisite to the balancing of the body with steadiness, and to the prevention of vertigo, may be diminished or destroyed in various ways, all of which seem to throw light upon this subject. Objects may become indistinct, 1st, by reason of their *smallness* and *similarity* to one another. Many persons become dizzy in a room hung with paper coloured with small and similar figures, where the eyes do not readily find a resting place, nor distinguish their movements in continually passing from one figure to another. But by affixing to the wall a sheet of white paper, or by drawing figures of a larger or more diversified size, the giddiness becomes no longer perceptible. It is for the same reason that vertigo is produced, in some, by passing over a plain covered with snow, without trees or other eminent objects. 2. Objects become indistinct, and the beholder vertiginous, on account of their *distance*, and the *direction* in which they are seen. It is for this reason that many become giddy in ascending lofty heights, or in looking down a deep precipice. Objects, placed at such a distance, are beyond the sphere of distinct vision, and, therefore, unsuitable to regulate our perpendicularity. The debilitating impression of fear must likewise be admitted, in this case, to produce a share of the effect. 3. The distinctness of objects is lost, and giddiness produced, by their *unusual* and *excessive motions*. Instances of this sort are very numerous, such as the view of a great cataract, of a large revolving wheel, &c. the first attempts to ride on horseback, to mount mount a camel, an elephant, &c. riding backwards in a coach, swinging, riding in a sleigh, skaiting, turning swiftly round on one foot, and more especially in the disease now under consideration.

The effect of these motions upon the organ of sight is also much increased by the *ocular spectra* of objects remaining some time upon the retina, which exceedingly augment the disturbance of the eyes, and thereby add to the confusion of the vertiginous person. When any one turns rapidly round till he becomes giddy, and falls upon the ground, the spectra of circumambient objects continue to present themselves in rotation, and he seems to behold such objects still in motion. These spectra appear to be a continuation of the motions of the optic nerve, which had been excited by the objects which they severally represent. They are apt to remain, to recur, or to be prolonged, in proportion to the degree of debility induced; hence they must greatly aggravate the more violent cases of sea-sickness, and produce an infinite number of deceptions of the sight and of imagination. Their effects are well known in fevers of debility, by producing the symptom called *muscæ volitantes*, &c.

Besides the vertigo of disordered vision, it is probable sea-sickness is generally produced, in part, by another species—that of disordered touch or feeling—and which has been called *tangible vertigo.* When a blind person turns round, or when one who is not blind revolves in the dark, a vertigo is produced belonging to the sense of touch: for his feet now touch the floor in manners or directions different from those they have been accustomed to; and, in consequence, he becomes bewildered as to the situation of his body in relation to the floor, loses his perpendicularity, and is rendered giddy. This combination of visual and tangible vertigo, in producing the phenomena of sea-sickness, seems to have escaped the attention of those who have treated of this disease. Sailors remark, that such persons as can soonest accommodate themselves to the ship's motion, and acquire the habit of standing and walking uprightly, without reeling to and fro, are least distressed by sea-sickness, and most speedily recover. The instability of visible objects, and the reeling induced in the beholder, reciprocally increase one another.

Having thus mentioned some of the various modes in which vertigo may be produced by a disordered and excessive action of the organ of vision and of touch, particularly such as arise from the rotation, undulation, or other irregular irregular and unusual motions of external objects, as well as of the beholders, I am, in the next place, to show in what manner vertigo produces the nausea and vomiting which quickly ensue.

It does not appear, at first view, how nausea and vomiting proceed from a disturbance of the action of the visual organ. But when it is recollected that violent giddiness, the immediate result of such disturbances, precedes and occasions these perversions of the alimentary canal, the difficulty vanishes. Vertigo, and disorders of the alimentary canal, reciprocally produce each other. Professor Gregory, of Edinburgh, asserts this in the following words: *Vertiginem nausea solet comitari, alteraque alteram inducere.*" * It is not the present object to enquire into all the species and varietes of vertigo which may be found enumerated in systems of nosology; but whether accompanying the attack of apoplexy, palsy, epilepsy, hysteria, or syncope; whether induced by injuries of the head from external violence, by excessive evacuations, or at the accession of fevers, it is generally attended with sickness of stomach. And, on the other hand, when the alimentary canal is primarily disordered, as in cases of indigestion, taking emetics, drunkenness, swallowing of poisons, gastritis, enteritis, &c. vertigo is generally found to take place. Sea-sickness is, therefore, a consequence of certain sympathies, or associations of motions of different parts of the animal system. And there is ground to conclude, that the vomiting caused by a stone in the bile-duct or in the ureter, as well as that arising from inflammation of the intestines, or at the accession of fevers, is produced in a similar manner.

If it be admitted that certain organs, or parts of the body, become associated in their actions (and the proofs of such an association continually recur in observing the functions of the animal system,) it will follow, that, in a state of health, each organ, or part, in this associated series or circle, has its appropriate share of nervous or vital power. But if one of these organs or parts be subjected to violent or irregular action, as such action consists in the employment and expenditure of nervous power, the balance of the distribution of this power must be disturbed, and while one part expends too much, the others will possess too little. This is obviously illustrated by the appearances

* Conspectus Medicinæ Theoreticæ, vol. i. p. 145.

ances of drunkenness. While the stomach is stimulated to excess by fermented or distilled liquors, the muscles of voluntary motion, the optic nerves, &c. are deprived of their share of nervous influence; and hence the inebriate becomes vertiginous, and his limbs refuse their accustomed office. Just so it is with persons unaccustomed to the motions of the water, when they go on shipboard. The excessive, irregular, and unusual actions of the organ of sight, expend a disproportionate share of nervous power, and, of consequence, the parts connected with it by association must soon suffer by a deprivation of their proper quantity. The stomach, which possesses more extensive and intimate relations with the rest of the system than any other viscus, will be the first to feel, and afterwards to propagate this morbid impression to other parts of the body.

Attention to the following circumstances will go far to explain the seeming disproportion between cause and effect, in this mode of accounting for the violence of sea-sickness in persons unaccustomed to the instability of that element. 1. The motion is not only unusual, irregular, and complicated, but excessive. The movements of the waves, forming a vast expanse of surface, agitated and rolling in a thousand shapes—the diversified movements of the ship, with all its variety of parts and appurtenances—and the movements of the voyager himself reeling and staggering in every direction: all these form an aggregate of agitation sufficient to distract the steadiest head. The contrast between this scene and such as are found on the land, where a great majority of objects are either at rest, or moving with steadiness and regularity, must be apparent to all. 2. The excessive action of the organ of sight, produced by this aggregate of unusual motion, will not appear strange if the quantity of nervous power expended on the eyes be duly considered. No part of the system, in proportion to bulk, is so plentifully supplied with nerves as the eyes. Each optic nerve is as large as a crow-quill at its entrance into the eye. Besides these, the third, fourth, and sixth pairs of nerves, as well as part of the fifth pair, belong to this organ. The incessant employment and activity of vision, during the day, considered in connection with the size and number of the nerves devoted to this sense, will evince that the consumption of nervous power in the eyes is fully as great, if not greater, than that bestowed upon the whole of the upper extremities. 3. The intimate sympathy between the brain and the the stomach is also to be considered; whereby the disordered actions of the organ of vision, which possesses so large a branch of the nervous system, make an immediate and powerful impression on the stomach, invert its motions, cause profuse discharges of bile, &c. and produce all the train of distressing sensations which belong to sea-sickness.

It is surprizing to observe what slight causes will, in some constitutions, produce vertigo. An unusual posture, an inconsiderable elevation from the ground, and even a momentary view of objects moving, so as to attract the gaze of beholders, will sometimes excite this sensation. Small modifications of motion will also serve to relieve as well as to produce it. A lady informed me, that, after constantly suffering nausea for some time, from riding in a sleigh, she was relieved, in the latter part of the journey, by a more harsh and rugged motion, in consequence of the snow suddenly dissolving, and leaving the earth bare. In this case, it is probable that the smooth and almost imperceptible progress of the sleigh, while gliding over the snow, prevented the lady's distinguishing the apparent motions of objects which were absolutely at rest, from the real motions of them; and this confusion seems to have been, at least in part, the cause of her giddiness and nausea. The course of the sleigh over sand, gravel, &c. was a nearer approach to ordinary habits of motion.

It is fortunate for such as are destined to a sea-faring life, and to other employments which are apt to produce similar giddiness and nausea, that these affections are commonly of short duration. The dominion of habit in these cases is extremely favourable. The Dervises of Turkey, who practise the motion of turning themselves swiftly round as a ceremony of religion, soon learn to perform it without giddiness. A similar habit is acquired among the Shakers, a fanatical sect of religionists in the State of New York. My colleague, Dr. Mitchill, informs me, that he saw a female of that sect turning herself round about sixty times in a minute, for the space of five minutes without interruption; and this was done without any appearance of her becoming vertiginous.

A question naturally arises on this subject, why some persons are more liable than others to vertigo and nausea, in consequence of unaccustomed motions. This is the result of a greater promptitude, in some constitutions, to run into sympathetic or associate actions. It is not easy to assign the reason why movements of the animal system, which have once occurred in succession or combination, should should afterwards acquire a tendency habitually to succeed or accompany each other. It is a property of animated nature, and distinguishes this department of being from others. There seems to be a peculiar temperament, consisting in the too great facility with which fibrous motions acquire habits of association, and in the strength with which these associations are maintained. In constitutions of this sort, sympathy acts with more certainty and energy, and to much greater extent, than in others. And it is probable, that such persons are much more liable than common to all the class of sympathetic diseases. For example, it might be expected that such would be peculiarly disposed to the attack of intermittent fevers; that the periodical return of paroxysms, in these cases, would be more difficult to arrest; and that they would be liable to recur, from slight causes, for many weeks after they had appeared to be cured. The force of memory seems also to depend upon the possession of this temperament; for memory is understood to mean facility and strength in forming and retaining associations. It would be matter of curiosity to ascertain, whether persons of retentive memory are more liable to fevers, to sea-sickness, and to all the various diseases of association, than others.

TREATMENT OF SEA-SICKNESS.

Having thus endeavoured to describe the appearance of Sea-sickness, and to assign the more probable causes, I proceed, in the next place, to the treatment of the disease.

Much may certainly be done towards the prevention of this disorder. It has been proposed, that persons intending to go to sea, should, for some time previously, accustom themselves to swinging; or to some other unusual motions adapted to induce giddiness. The exercise of turning round upon one foot, would probably answer this purpose as well; and it may be acquired, after some practice, so as to be performed entirely without vertigo.

( To be continued. )

